# The Naphthols as Intestinal Anti-Infectants

**Published:** 1893-07-01

**Authors:** George Parker

**Affiliations:** Assistant Physician, Bristol General Hospital


### THE NAPHTHOLS AS INTESTINAL ANTI-INFECTANTS.

By GEORGE PARKER, M.A., M.D. Cantab.,
Assistant Physician, Bristol General Hospital.

The aromatic series of carbon compounds is perhaps the most fertile source of disinfectants which we possess. Carbolic, salicylic, benzoic acids, resorcin, thallin, cresol, thymol, and naphthaline are all modifications of benzene $C_6$ $H_6$. By the union of two benzene groups we get naphthaline $C_{10}$ $H_8$, and from this two isomeric compounds the alpha and beta naphthols. These are all powerful antiseptics of tried value, and members of the same series. The theories of the great Bishop Berkeley on the virtues of tar water turn out to be of equal importance with his theory of vision, and his defence of idealism. The naphthols, derived from tar, have besides their antiseptic action on lowly organisms this peculiarity that they produce little or no poisonous effect on the higher animals, because they are so insoluble that even when taken internally they are not absorbed into the system.

They have been found very useful in the vomiting and diarrhœa of children, in summer and tubercular diarrhœa, in typhoid, and externally in scabies, eczema, and other skin diseases.

Two or three grains of either of the naphthols are given every two hours for twelve or twenty-four hours in diarrhœa, with the result of rendering aseptic the contents of the intestinal canal. The effect in cases where much flatulence and offensive slimy stools are present is very marked. The napthols have not the unpleasant odour of naphthaline, but they are somewhat hot and pungent to the tongue. As an ointment containing 10 per cent., they can be used for skin diseases. Their most important application, perhaps, is in typhoid fever. Now, we know of no antiseptic which can be taken in sufficient quantity to render the blood and entire tissues uninhabitable by the typhoid germ, and so cut short the disease at once. Dr. Burney Yeo, indeed, administers chlorine water as a general disinfectant, and he claims to have had results of some value, but the evidence is not conclusive. To look for any action of this kind from the naphthols would only lead to disappointment. However, a large part of the evils in typhoid arise from secondary infection of the ulcerated surface of the bowels by putrefactive organisms and absorption of their products. Recent treatment has been directed largely to prevent this. It was necessary to find some remedy which would not be absorbed before the small intestine was reached, and the two drugs, salol and naphthol, fulfil this indication. Salol passes through the stomach uninfluenced by the acid gastric juice, and is broken up in the small intestine into carbolic and salicylic acids, which act as disinfectants until absorbed. Naphthol, though antiseptic, is insoluble, and therefore passes along the whole course of the canal. Four questions at once suggest themselves. Does napthol destroy the typhoid bacillus when brought into contact with it? Secondly, if it has this power, does it affect the bacilli which lie deep in the glands? Does it destroy the putrefactive organisms, and prevent secondary poisoning? Lastly, does it interfere with normal fermentations, and the ordinary processes of digestion?

Now, the typhoid bacillus, though growing in most media, either in the body or without, and even in earth, does not seem to have great resisting powers, and there is not much doubt that sufficient hydro-or B-naphthol can be given to destroy it when lying in the intestinal canal.

However, as to the second question, it is abundantly clear that the advocates of naphthol cannot hope to reach the germs in the tissues. Roux has shown typhoid bacilli from the spinal canal, and from an abscess of the spleen in a typhoid case where even the Peyer's glands where not swollen; Ebermeyer isolated them from the medulla of bones, and Colzi from a thyroid abscess in typhoid patients. It is evident from this that we cannot do more, as regards the typhoid bacilli, than prevent fresh colonies entering the system, and save the intestines from commencing ulceration. On the other hand, the already existing ulcers are, as Ruffer says, an open door through which staphylococci or other organisms of suppuration enter to produce many of the abscesses found in typhoid. These can be and ought to be prevented by naphthol continuously administered.

In answer to the fourth question Dr. J. Michell Clarke has proved that hydro-naphthol exercises only a very slight retarding influence on the digestion of milk by the stomach, and has no effect on pancreatic digestion. He considers that in the majority of cases it "does not interfere with the normal fermentative processes, or give rise to digestive disturbances," but he mentions two instances where this occurred, and where the drug had to be stopped for a time. Still it is rare for any irritation or vomiting to be set up. Most patients take it easily and find relief from distension, diarrhœa, and cerebral troubles. The pungent taste which children especially dislike is not noticed, as the tongue and palate in typhoid have lost most of their activity.

On the whole, then, we may conclude that naphthol may reduce the number of the invading bacilli: it can prevent secondary pyæmia; and it rarely interferes with digestion, but it cannot touch the bacilli which have passed through the intestinal wall. A corollary from the proved immunity of the deeply seated bacilli may be laid down, that it cannot prevent relapses or hinder the pyrexia due to the action of those bacilli, and this is found to be the case in practice. The value, then, of the naphthol treatment lies in this, that the diarrhœa is cut short and the stools become inoffensive, the distension, together with abdominal pain and tenderness, quickly disappears, the secondary fever is cut short, the tongue cleans, convalescence is more rapid, and, so far as clinical experience has gone, secondary complications with hæmorrhage and perforation are made rare or altogether prevented. Naphthol can be given in cachets, or suspended in mucilage or milk, or simply as a powder. The dose for an adult is two or three grains every two hours, which may be continued for weeks with the effect of keeping the intestinal tract aseptic. If the patient objects to the taste we may employ benzonaphthol, which is recommended by Yvon and Gilbert as tasteless and odourless, giving twelve doses daily of four to six grains. This substance breaks up in the intestine into B-naphthol and benzoic acid, and acts after the manner of its components. With this treatment, the value of which has been shown by the reports of numerous investigators, and by continued use in the Bristol General Hospital and elsewhere, we can combine advantageously other remedies. Cold baths have been extolled by some and decried by others. They not only reduce the temperature, but cause the toxins to be rapidly thrown out of the system, yet the objections to their use are very weighty. We can keep down high temperatures by quinine, phenacetin, and antipyrsin—quinine, indeed, unless ear troubles are present, has a special value—or the cold air method may be adopted, but the pyrexial period is usually a short one if naphthol is continuously administered.

In conclusion we must bear in mind that naphthol is no specific for the disease, but it fulfils, without causing any fresh risks, a rational indication. No surgeon would leave large ulcerated surfaces exposed to septic influences, and the administration of the one drug which acts on them and only on them in typhoid has been abundantly justified by the mitigation of the general symptoms which follow its use.

